# Hyperhomocysteinemia: Focus on Endothelial Damage as a Cause of Erectile Dysfunction

**DOI:** 10.3390/ijms22010418

**Published:** 2021-01-03

**Authors:** Gianmaria Salvio, Alessandro Ciarloni, Melissa Cutini, Giancarlo Balercia

**Affiliations:** Division of Endocrinology, Department of Clinical and Molecular Sciences, Polytechnic University of Marche, via Conca 71, Umberto I Hospital, 60126 Ancona, Italy; g.salvio@pm.univpm.it (G.S.); a.ciarloni@pm.univpm.it (A.C.); m.cutini@pm.univpm.it (M.C.)

**Keywords:** Erectile Dysfunction, hyperhomocysteinemia, endothelial dysfunction

## Abstract

Erectile Dysfunction (ED) is defined as the inability to maintain and/or achieve a satisfactory erection. This condition can be influenced by the presence of atherosclerosis, a systemic pathology of the vessels that also affects the cavernous arteries and which can cause an alteration of blood flow at penile level. Among the cardiovascular risk factors affecting the genesis of atherosclerosis, hyperhomocysteinemia (HHcys) plays a central role, which is associated with oxidative stress and endothelial dysfunction. This review focuses on the biological processes that lead to homocysteine-induced endothelial damage and discusses the consequences of HHcys on male sexual function

## 1. Introduction

Erectile Dysfunction (ED) is defined as the persistent inability to obtain or maintain penile erection sufficient for a satisfactory sexual performance [1] and represents a common condition in middle-aged men, with a prevalence that increases exponentially with age. According to data from the Massachusetts Male Aging Study (MMAS), 52% of men between 40 and 70 years old report some form of ED with a percentage that increases proportionally with aging and reaches 70% at 70 years of age [2]. ED has a multifactorial etiology with arteriogenic, neurogenic, endocrinological or iatrogenical causes, but most cases of ED are determined by impaired penile blood flow. Many medical conditions (e.g., diabetes mellitus, hypertension, obesity) are associated with ED and the strong association between ED and the development of cardiovascular disease (CVD) was already evident in the late 1990s [1]. It is estimated that one in five ED patients may have asymptomatic CVD and the severity of ED is strongly correlated with the extent of CVD [2]. The lowest common denominator between CVD and ED is currently recognized as being endothelial dysfunction, whose fundamental mechanism is the reduced expression and activation of nitric oxide synthase (NOS) which is responsible for the formation of nitric oxide (NO), one of the most powerful vasodilators in the human body and which plays a fundamental role in the induction and maintenance of penile erection (see later) [3].

In recent years, homocysteine (Hcys) has gained the attention of numerous authors due to its possible pathogenetic role in endothelial dysfunction and, consequently, its possible involvement in the onset of CVD. The present paper aims to evaluate the role of Hcys in the development of ED, particularly as regards the physiopathological implications and molecular mechanisms underlying endothelial dysfunction. In addition, the benefits and ¬ways of treating hyperhomocysteinemia (HHcys) will be discussed in the light of the latest available evidence.

## 2. Homocysteine Metabolism and Hyperhomocysteinemia

Hcys is a sulfur-containing amino acid deriving from the essential amino acid methionine, which is abundant in animal proteins. It circulates in plasma in four different forms: free thiols (around 1%), disulphide-bound to plasma proteins (70–80%), Hcys-Hcys dimers and Hcys-other thiols dimers [4]. Hcys undergoes two different metabolic processes which follow opposite directions: trasnssulfuration and re-methylation. The first process is activated when methionine is introduced in excess with diet and leads to the transformation of methionine into Hcys by the enzyme cystathionine beta synthase (CBS), whereas the latter occurs when levels of methionine levels are low and leads to the conversion of Hcys by the enzyme methionine synthetase, with vitamin B12 as a cofactor, and by the enzyme 5,10-methylenetetrahydofolate reductase (MTHFR), with folic acid (FA) as a cofactor. Through these enzymatic pathways, serum Hcys levels are maintained in a safe range with constant turnover. HHcys can occur when the re-methylation pathway of Hcys is defective due to congenital (e.g., genetic defects of MTHFR) or acquired conditions, the latter including vitamin deficiencies (primarily B6, B12 and FA) and renal impairment [5].

HHcys is a clinical condition defined by abnormally high Hcys levels in serum, which correspond to values greater than 15.0 μmol/L or 12.0 μmol/L when assessed with the use of high-performance liquid chromatography or immunoassay methods, respectively. Severe forms of HHcys are commonly due to major genetic mutations of the enzymes implicated in the Hcys metabolism (e.g., MTHFR polymorphisms, see later), whereas slightly elevated Hcys values are more frequently caused by vitamin deficiencies and environmental factors [4].

Genetic defects may be present at any level of the Hcys metabolic pathway and lead to various medical conditions. One of the most common is the CBS defect, which occurs in approximately 1 of 100,000 live births, compromises the transsulfuration pathway and determines the clinical picture of classical homocystinuria, which results in an up to 40-fold increase in fasting Hcys. When untreated, this condition is accompanied by the onset of a vascular event (stroke, myocardial infarction or other thromboembolic complications) in about half of the patients before the age of 30. Mutations of MTHFR and of the enzyme methionine synthetase, on the other hand, determine a variety of diseases called “disorders of remethylation”, which are multisystem pathologies dominated by neurological and ocular disease and by failure to thrive, all of which are associated with high levels of Hcys [6]. Less severe mutations of MTHFR or the presence of certain polymorphisms may determine the appearance of HHcys in the absence of other syndromic manifestations [7].

On the contrary, common acquired conditions that can increase Hcys levels are nutritional deficiencies of vitamin B6, vitamin B12 and FA, as demonstrated by the inverse correlation between blood levels of these vitamins and serum Hcys. A slight increase in Hcys serum levels can also be observed in several conditions such as nephropathy, thyroid disease, cancer, psoriasis, diabetes, older age and menopause. Tobacco, coffee and certain drugs interfere with the folate metabolism and may also raise Hcys levels. Conversely, physical activity and moderate alcohol consumption are associated with lower Hcys levels [4].

Regardless of the cause, the increase in Hcys levels is of considerable clinical importance due to the high rate of cardiovascular complications to which it is associated. The causes and clinical manifestations of HHcys-related endothelial dysfunction will be explored in the following sections.

## 3. Hyperhomocysteinemia and Endothelial Dysfunction

The endothelium plays a pivotal role in the regulation of vascular wall homeostasis (e.g., regulation of vascular tone, coagulation and permeability) and the term “endothelial dysfunction” indicates a pathological process which represents the first step in the pathogenesis of atherosclerosis, a pathology that involves all the vessels of the body and which, in most cases, presents a progressive course. Endothelial dysfunction is usually triggered by an inflammatory insult that leads to the activation of endothelial cells which produce adhesion molecules and chemokines. The latter are responsible for the recruitment of circulating monocytes which, after having infiltrated the intima, are activated into macrophages that internalize modified lipoproteins and convert them into foam cells. At the same time, inflammatory molecules lead to a structural remodeling of the atherosclerotic lesion which becomes a fibromuscular plaque and further develops a fibrous cap with a lipid-rich necrotic core. The persistence of the inflammatory stimulus is responsible for the plaque’s instability and may contribute to its rupture, with luminal release of the thrombogenic core, and to the consequent atherothrombotic occlusion of the vessel involved [8].

Numerous clinical and experimental findings have shown that HHcys can alter the homeostasis of the endothelium and contribute to the development of atherosclerosis. In vitro, Hcys impairs the dilatory effect reacting with O_2_^−^ to form peroxynitrite (ONOO^−^) and reducing the bioavailability of NO, which exerts an important role as a vasodilator [9]. Moreover, Hcys increases endothelin-1 expression, which is a potent vasoconstrictor [8]. It goes without saying that the sum of these alterations induces profound alterations in the homeostasis of the endothelium reducing its ability to regulate vascular tone. Recently, in a study on a very large population (over a thousand patients), a significant association was demonstrated between HHcys and coronary microvascular endothelial dysfunction (which represents a feature of early atherosclerosis) evaluated with functional angiography. After adjustment for classical CVD risk factors (age, sex, body mass index, chronic kidney disease, diabetes mellitus, smoking exposure, low-density lipoprotein cholesterol, high-density lipoprotein cholesterol and triglycerides and aspirin, statin, and B vitamin use), the authors reported that being in the highest quartile of serum Hcys level (>9 µmol/L) was an independent predictor of coronary microvascular endothelial dysfunction with a 1.34 OR (*p* = 0.03) [10].

Moreover, HHcys indirectly exerts a further action on the endothelium causing an increased release of reactive oxygen species (ROS), a family of molecules which in turn damages the integrity of the endothelium and favors the activation of endothelial cells [8]. In addition, at a later stage of the atherosclerotic process, Hcys increases platelet activation and aggregation, promoting vascular occlusion where endothelial activation is already present [11].

## 4. Hyperhomocysteinemia and Cardiovascular Disease

As previously illustrated, the presence of high Hcys levels in serum is accompanied by endothelial dysfunction, which represents the pathophysiological basis of atherosclerosis. The clinical manifestations of the latter are represented by CVD, which is the effect of reducing the blood flow to the level of the main organs of the body (eg heart, brain, kidneys and limbs). The first hypothesis on the link between Hcys and CVD dates back to the 60s with what at the time was called “the Hcys theory of arteriosclerosis” [12], but HHcys was officially recognized as a risk factor for CVD only thirty years later [4]. Since then, three different pathophysiological mechanisms have been proposed to explain this relationship: (1) the reduction, by Hcys, of the bioavailability of NO at endothelial level, (2) the direct cytotoxic effect of Hcys mediated by oxidative stress, (3) dysregulation of the folate metabolism [12].

Numerous findings have shown a strong association between Hcys levels and the clinical manifestations of atherosclerotic diseases such as stroke, peripheral artery disease, carotid artery disease and chronic kidney disease. In particular, HHcys is accompanied by a worsening of the outcome of all these conditions, especially in the presence of other cardiovascular risk factors: for example, increased Hcys concentrations are a risk factor for ischemic stroke in hypertensive patients, whereas in diabetic patients HHcys is associated with the development of microvascular complications such as retinopathy, neuropathy and nephropathy [13]. An increase of 5 µmol/L Hcys in serum is associated with a 1.6 odds ratio for coronary artery disease, whereas it has been suggested that HHcys correction could lower the risk of ischemic heart disease and stroke, even if intervention studies have provided conflicting results [5]. Interestingly, high Hcys levels in serum have been found in patients with migraine compared with healthy controls. Since migraine is an important risk factor for stroke, it has been supposed that the link between these conditions could be a state of hypercoagulability [14].

Levels of Hcys in serum have also been associated with different indices of arterial stiffness such as pulse pressure and aortic stiffness assessed by carotid-femoral pulse wave velocity. The latter has proved to be higher in HHcys patients compared with the general population with a significant correlation in a linear regression analysis model [4].

Despite the strong association between the two conditions, the question remains whether Hcys represents only a biomarker (an epiphenomenon from CVD) or a true cardiovascular risk factor, therefore it is still a matter of debate whether the correction of HHcys can bring about a reduction in the incidence of cardiovascular events (see below). What is certain is that ED, given its vascular nature in most cases, is closely linked with CVD, and that all cardiovascular risk factors, including Hcys, represent an important element in the assessment of male sexual dysfunction.

## 5. The Molecular Mechanisms of Homocysteine-Related Erectile Dysfunction

Penile erection involves neurogenic, psychogenic and hormonal mechanisms that ultimately involve NO as the main mediator through the stimulation of non-adrenergic, non-cholinergic nerves. NO is released by the endothelium and neurons in penile tissue and binds to soluble guanylyn cyclase to increase the production of 3′,5′-cyclic guanosine monophosphate (cGMP), which activates protein kinase G (PKG) to form a complex cGMP/PKG that induces the relaxation of smooth muscle in the corpora cavernosa, with a consequent hyper-flow of arterial blood which represents the fundamental mechanism of penile erection [15]. Damage to endothelial cells, caused by numerous conditions such as hypertension, smoking and diabetes, leads to a reduction in NO release in the corpora cavernosa thereby favoring the development of vasculogenic ED. Phosphodiesterase type 5 (PDE5) inhibitors (e.g., sildenafil) act by blocking the degradation of NO in the corpora cavernosa and they are the first line of treatment for ED and are effective in about 70% of cases [1]. NO is generated by an enzyme called NO synthetase (NOS) present in three isoforms: endothelial NOS, neuronal NOS and inducible NOS (eNOS, nNOS and iNOS, respectively). Among these, low eNOS and nNOS activity has been linked to ED in animal models by several studies [16] and low eNOS activity has also been observed in conditions such as hypercholesterolemia, diabetes and advanced age [17].

Studies in animal models have shown that HHcys can cause ED in several ways. Firstly, as already discussed in the previous section, Hcy directly causes atherosclerosis acceleration, hence impairing endothelial repair capacity, promoting platelet activation and dysregulating the lipid metabolism [13]. In this way, among others, atherosclerosis, being a systemic pathology, involves the cavernous arteries of the penis, through which the blood flow is reduced. Montorsi et al., in 2005, first described what is known as “the artery size hypothesis”, suggesting that the clinical manifestations of atherosclerosis depend on the caliber of the affected vessel and that, due to the progressive nature of atherosclerotic disease, the persistence of a pathogenic noxa at endothelial level increases the number of organs involved over time [18]. Given the small caliber of the cavernous arteries, it is clear that ED will be the first manifestation of atherosclerosis in the vast majority of vasculopathic patients.

In addition to determining structural alterations in the vessel wall, HHcys may also compromise the homeostasis of the endothelium in a functional way interfering with the metabolism of NO. In vitro, HHcys inhibits acetylcholine-induced relaxation and cGMP production in the rabbit corpus cavernosum [9], whereas, in vivo, it decreases eNOS expression in the inner wall of both the cavernous sinus and the vessels and affects the number of erections and latency after apomorphine injection [19]. In addition, in the corpora cavernosa, HHcys determines the reduction in smooth muscle cells and the expression of endothelial cell junction proteins (Ve-cadherin, occludin and caludin-5) and eNOS and an increase in the degree of fibrosis [17].

HHcys can act in a further peculiar way. Khan et al. were the first researchers to demonstrate that Hcys can induce ED not only by inhibiting NO formation but also through the generation of toxic free radicals. They reported that Hcys inhibits carbachol-stimulated relaxation (a NO-dependent mechanism) of cavernosal smooth muscle strips of rabbit. This inhibition is further increased by copper administration, that augments the oxidation of Hcys to generate H_2_O_2_ and catalyzes the subsequent reaction which produces ROS. On the contrary, the inhibition of NO-dependent relaxation of the corpus cavernosum is reversed by catalase or superoxide dismutase, which instead accelerate the elimination of ROS (catalyzing the transformation of O_2_^-^ into water) [9]. On the basis of these observations, ROS has been proposed as the main mediator of homocysteine-induced endothelial damage. In a recent review, Kaplan et al. extensively investigated the relationship between ROS and Hcys. They reported that studies of mitochondria isolated from different tissues provided conflicting results. In particular, studies on samples of cerebral cortex and hippocampus of rats treated chronically with Hcys showed an increase in mitochondrial ROS production, while the opposite effect was observed in neuronal cells after acute administration of high doses of Hcys. Furthermore, chronic treatment with medium doses of Hcys showed no significant effects in terms of oxidative stress on either heart or kidney. This variability could be explained by the presence of several molecular mechanisms involved in ROS production by Hcys, such as auto-oxidation (with production of O_2_^-^ and H_2_O_2_) and N-homocysteinylation of mitochondrial proteins, but the available evidence is insufficient to draw firm conclusions [20]. Overall, there is a great body of evidence supporting a powerful modulatory effect of Hcys on the cellular oxidative system. Clarifying the nature of this relationship could be the key to developing therapeutic strategies to prevent the vascular complications of Hhcys.

## 6. Hyperhomocysteinemia and Erectile Dysfunction: Clinical Evidence

Considering the experimental evidence, it is legitimate to ask whether HHcys can represent an important clinical finding and what its real impact is on male sexual function.

In a large study, Hcys, FA and B12 levels were recently assessed on a total of 1381 patients (688 ED and 693 non-ED). The authors reported a significant association between Hcys and erectile function, especially in older men (age ≥ 60), with a larger proportion of HHcys in the ED group than the non-ED group (43.02% versus 37.52%, *p* = 0.037) [21]. In particular, Demir et al. demonstrated that HHcys increases more than three times the risk of ED [22].

In a recent meta-analysis conducted on nine different studies, Sansone et al. confirmed that HHcys is more often observed in subjects with ED rather than controls, with a standardized mean difference of 1.00 (95% CI 0.65–1.35, *p* < 0.0001). Moreover, in ED patients, they observed that subjects without diabetes are more likely to have increased levels of Hcys than diabetic subjects [23]. This is in contrast with previous studies [24,25] and HHcys has been associated with ED in several other medical conditions such as, for example, hepatitis C virus infection [26]. However, even if it could seem counter-intuitive, some authors suggest that in the presence of comorbidities such as diabetes, even small changes in serum Hcys could negatively affect erectile function acting in a synergistic way and becoming clinically relevant [23].

The human MTHFR gene is localized in the short arm of chromosome 1 and, as previously mentioned, represents the key enzyme in the remethylation of Hcys. Two common MTHFR polymorphisms (C677T and A1298C) are both associated with decreased enzyme activity and consequent increasing serum levels of Hcys that lead to endothelial dysfunction, CVD and the early onset of vasculogenic ED. In particular, young adult individuals (mean age 32.2 years) with the MTHFR 677TT genotype and the 677TT + 1298AC combined genotype were found to have a 3.16 and 3.89 fold increased risk of developing ED compared to age-matched healthy controls [7]. This is particularly interesting especially if we consider that the patients affected by this anomaly are young and lack classical cardiovascular risk factors and therefore, based on clinical-anamnestic data alone, the vascular cause of ED could be erroneously excluded. Confirming this, in a group of ED patients without vascular risk factors and with normal vascular parameters according to penile doppler ultrasonography, higher Hcys levels were observed compared with healthy cases, especially as regards patients who were non-responders to sildenafil treatment [27].

Since FA acts as a cofactor in the remethylation process of Hcys [5], its role in the development of ED is also currently being studied. Folate deficiency may derive from inadequate dietary intake, medications (e.g., metothrexate and anticonvulsivants) and alcoholism. It has been hypothesized that FA deficiency could play a central role in ED development both by directly interfering with the NO metabolism and by favoring the development of HHcys. Some authors [3,28,29,30] consider low serum FA levels as a risk factor for ED whereas others do not agree [21]. Sansone et al. reported significant lower serum FA levels and higher serum Hcys levels in ED patients compared to non-ED patients, even if, surprisingly, these authors did not find a significant correlation between Hcys and FA levels [28]. Attia et al. further reported that low serum levels of FA are an independent risk factor for ED and that a cut-off value of 9.42 ng/mL is able to detect ED patients with a sensitivity of 80.0%, a specificity of 93.3% and an area under curve (AUC) of 0.913. Moreover, they found a negative correlation between serum FA and the severity of ED that remained significant after adjustment for diabetes, hypertension, smoking, age and cholesterol [3].

In conclusion, it is clear that HHcys represents an important risk factor for ED and its value increases especially in those patients in whom the origin of sexual dysfunction cannot be identified only on the basis of classical cardiovascular risk factors.

## 7. Hyperhomocysteine-Lowering Intervention and Erectile Dysfunction

Although the available evidence has amply demonstrated the association between CVD and Hcys levels, it is still a question of debate whether the correction of HHcys represents a beneficial therapeutic goal and can improve cardiovascular outcome in these patients. Moreover, the optimal way to treat HHcys has yet to be determined since several methods of treatment have been described as reducing Hcys levels. A recent Cochrane review investigated the effect of B-complex vitamin (cyanocobalamin or B12, FA and pyridoxine or B6) administration as Hcys-lowering intervention for preventing cardiovascular events. Data were extrapolated from 15 randomized controlled trials involving 71,422 participants and showed no reduction in the incidence of either myocardial infarction or death from any cause, although the authors found a reduction in the risk of stroke with no differences, compared to controls, in terms of side effects. Based on these results, the review concluded that there is still the need for large co-operative trials in order to compare types of Hcys-lowering intervention so as to identify the optimal dosage and possible association with other medications [12].

As regards ED, lifestyle modifications are considered the first-line therapy. Subsequent treatment strategies include PDE5 inhibitors, testosterone replacement treatment, intracavernosal injection therapy, vacuum constriction devices, intraurethral prostaglandin suppositories and surgical placement of a penile prosthesis [1]. Among these, PDE5 inhibitors have the advantage of exerting cardioprotective properties [31], which, in addition to the excellent safety profile and their ease of intake, makes them the first choice when a pharmacological treatment is required. PDE is a family of enzymes modulating the intra-cellular concentration of cyclic adenosine monophosphate (cAMP) and cGMP through their degradation. PDE5 inhibitors act by increasing the NO-dependent production of cGMP in cavernous vascular smooth muscle cells, thus facilitating penile erection, but they also have beneficial cardiovascular effects, improving cardiac output, remodeling cardiac muscle cells, inhibiting platelet aggregation and reducing the production of inflammatory molecules such as IL-6 [32].

As already discussed, MTHFR mutations are an important cause of HHcys. The treatment of a group of ED patients with the MTHFR 677TT genotype using a combination of sildenafil, FA and vitamin B6 led to a significant reduction in Hcys levels (*p* < 0.001) and a marked improvement in the PDE5 inhibitors treatment response rate. In addition to confirming the importance of the MTHFR genotype in the development of ED, these data suggest that mutated MTHFR activity is modulated on the basis of folate availability and that a correction of low FA levels may help correct the HHcys which determines ED and also improve the subsequent response to treatment with sildenafil or with molecules of the same class [33].

In a previous study, 83 patients took a combination of tadalafil (10 mg every other day) and FA (5 mg daily) or tadalafil and placebo for three months. Although both groups showed a significant improvement in sexual function after treatment, shifting from moderate ED to mild-moderate ED, in the FA group the post-treatment IIEF score was significantly higher than in the placebo group (16.80 vs. 14.37, *p* = 0.002) [34].

FA may exert a positive action on endothelial function and penile erection by decreasing serum Hcys levels, although recent evidence suggests that it could act directly on the NO metabolism thereby improving the efficiency of NOS [19]. Moreover, FA may also improve penile blood flow by preventing the platelet aggregation caused by Hcys [11]

A recent study by Elshahid et al. was conducted on 50 patients affected by vasculogenic ED and 50 healthy controls who underwent FA administrations (500 mcg per day for three months). These authors reported that FA supplementation significantly improved sexual function both in terms of IIEF-5 (the median score increased from 6 to 14, *p* < 0.001) and penile doppler evaluation (the median peak systolic velocity increased from 21.45 to 27.6, *p* < 0.001), concluding that FA is a potential safe drug that could be prescribed in ED patients, alone or in association with PDE5 inhibitors drugs to improve erectile function [5].

Recently, Zhang et al. have proposed a treatment with a demethylation agent, 5-aza-deoxycytidine, in a model of HHcys-related ED. These authors assumed that the hypermethylation of the promoter of the gene encoding the dimethylarginine dimethylaminohydrolase protein could play a key role in ED development, since it metabolizes asymmetric dimethylarginine, which is an endogenous inhibitor of NOS. They reported that six weeks of 5-aza-deoxycytidine administration in methionine-fed rats significantly increased NO and cGMP levels (*p* < 0.05) and the intracavernous pressure/mean systemic arterial pressure ratio (*p* < 0.05), providing a novel therapeutic method [35].

A promising alternative could be represented by the activation of the kallikrein-kinin system. Tissue kallikrein-1 is a glycoprotein that processes kininogen to produce vasoactive kinins, which exert a number of beneficial biological effects especially at endothelial level. In an animal model of HHcys-induced ED, Cui et al. recently reported that transgenic rats harboring the human tissue kallikrein-1 gene exhibited better sexual function than wild-type rats [17]. However, it remains to be clarified whether these results can also be applied to humans and what might be the method for applying this discovery in clinical settings.

To sum up, although clinical data are limited, the evidence available so far suggests that, in the presence of HHcys-related ED, the administration of Hcys-lowering agents (e.g., FA and vitamins B6 and B12) could represent a good therapeutic choice in terms of risks and benefits. Moreover, it can be hypothesized that the simultaneous administration of PDE5 inhibitors and of Hcys-lowering agents may have a synergistic effect on the NO-dependent relaxation of smooth muscle cells and interrupt the progression of endothelial damage in the corpora cavernosa. Finally, preclinical studies have provided promising results on new types of treatment whose validity will also have to be confirmed in humans in the near future.

## 8. Conclusions

There is a growing body of evidence supporting the close correlation between HHcys and ED. The molecular mechanisms that link the two conditions involve NO metabolism and oxidative stress, which underlie endothelial dysfunction. Since the latter also represents the primum movens for the development of CVD, the interruption of the cascade of phenomena that from the first endothelial damage lead to atherosclerosis would represent an undoubted advantage. Therefore, further studies are needed to unambiguously establish the optimal pharmacological strategy in the context of Hcys- lowering treatments in terms of safety and benefits.

## Abbreviations

ED	Erectile Dysfunction
CVD	CardioVascular Disease
NOS	Nitric Oxide Synthase
NO	Nitric Oxide
Hcys	Homocysteine
HHcys	HyperHomocysteinemia
MTHFR	5,10-Methylenetetrahydofolate Reductase
FA	Folic Acid
ROS	Reactive Oxygen Species
cGMP	3′,5′-cyclic Guanosine MonoPhosphate
PKG	Protein Kinase G
PDE5	PhosphoDiEsterase type 5
